# Panel Pay and Extended Services.—I

**Published:** 1923-05

**Authors:** R. W. Harris


### PANEL PAY AND EXTENDED SERVICES.—I.

**By R. W. HARRIS.**

I HEARD the other day of a critic who had for some time past been keenly supporting some sort of vague change in the relations of panel doctor and patient which would revolutionise the Insurance Medical Service, bring doctor and patient into the same sort of perfect relationship which is supposed to exist between doctor and private patient, cause complaints to disappear, and generally bring about a vastly improved state of affairs. He has now reached the conclusion that the whole thing turns on the question of finance. He has, in other words, arrived after a good deal of exertion at the starting point. It is satisfactory, at least, to learn that he has arrived.

### The Domination of Finance.

It is because of this dominating consideration of finance that it is useless to discuss at the present time anything in the nature of a complete medical service for the insured, and still less for their dependents. But if the question is asked whether any extension of the present service is not possible, I should venture to reply that it is not only possible, but very desirable, unless we are to have a continuance of the present discontent with the Insurance Medical Service. It is certain that that service will go on, in spite of confident forecasts in some quarters that the " panel system " is coming to an end this year. I have already, in a previous article, said that the Societies generally do not seek to control the doctors, and I am sure that we shall not see any demand of this kind pressed, if indeed it is put forward.

### The Panel Doctor's Pay.

Dr. Cox has recently pointed out that before the question of the pay of the panel doctor can be settled for the future it is necessary to determine the nature of the service to be required of him, or, in other words, whether there is to be any modification in the terms of the present contract, apart from minor matters. He has also pointed out that, after the end of this year, 7s. 3d. only per head of the insured population will be available for paying the panel doctor (who at present gets 9s. 6d.), unless other moneys are forthcoming from a special Exchequer grant—which the Government may be expected to resist; from a grant from funds which are, in effect, the funds of Approved Societies—which the Societies may be expected to resist; or from an increased weekly insurance contribution—which everybody may be expected to resist. If, therefore, the doctors will not accept 7s. 3d.—and there seems little likelihood of this—there arises a pretty problem for settlement in which those of us who are lookers-on may return thanks for not being concerned. (In passing, it seems doubtful if even 7s. 3d. can be said to be available. The total sum provided by the Act of 1920 for Medical Benefit is 9s. 6d. Out of this, drugs and appliances have to be provided, and also mileage for the rural doctors; the former is not a fixed yearly sum and for the two taken together it is probably not safe to reserve less than 2s. 6d. out of the sum of 9s. 6d., leaving 7s.). In circumstances such as these it is not helpful to say merely that something must be done or to make some reference to the wit of man. I should like to make one or two observations of a general character which may at first seem equally unhelpful but which, as it appears to me, need to be clearly stated and constantly borne in mind.

### Some Axioms.

A National Health Insurance Service without doctors is unthinkable. Public opinion will require that the doctors should be properly paid. What is a proper rate of payment is a question of great complexity which public opinion or sectional prejudice cannot settle and which can probably only be settled, so as to satisfy public opinion, by Arbitration. But when the amount is so settled it should properly form a first charge on National Health Insurance funds and should not be made the subject of special supplementary grants. The withdrawal of supplementary grants to which at present conditions are attached, giving the Minister effective control over the service, must not be accompanied by any weakening of that control; the conditions must, therefore, be transferred to the ordinary Exchequer contribution forming part of the normal Insurance funds. I think also that there will be general support for the view that the settlement to be made with the doctors should now be of a quasi-permanent character and expressed to operate for a term of five years at least.

### How to Pay for Extensions.

If there is to be a settlement of this permanent character does this mean that the present restricted service is to continue without any hope of extension, and is there any useful purpose to be served in discussing extensions (which, of course, have to be paid for) when the question of finding money for the present restricted service is already so difficult of settlement? Taking the latter point first I have observed that, at the Guildhall Conference when the subject of extensions of service was under discussion very tentatively, Sir Thomas Neill said that if the period of sickness benefit could be reduced by one day in the case of each person it would provide a substantial sum for extension of services. This idea seems well worth exploring; in particular it has the merit of providing a fund for the benefit of insured persons at their own expense, and it is not, therefore, open to the objection, sometimes heard, that further benefit should not be provided for one section of the community. An examination of the restrictions which are at present imposed on the Medical Service for the insured and of those extensions especially which may directly affect the panel doctor's contract, before his remuneration for the future is settled, must be reserved for another article.

---

It is announced that Westminster Hospital will close in June for six months for repairs.

